# Transcriptome sequencing leads to an improved understanding of the infection mechanism of *Alternaria solani* in potato

**DOI:** 10.1186/s12870-023-04103-3

**Published:** 2023-03-01

**Authors:** Jia Jiang, Xuhao Guo, Huanhuan Tan, Mingya Ding, Fangming Liu, Zhihui Yang, Jiehua Zhu

**Affiliations:** 1 College of Plant Protection, Hebei Agricultural University, Baoding, 071001 China; 2 Department of Plant Protection, College of Horticulture and Plant Protection, Henan University of Science and Technology, Luoyang, 471023 China; 3 Potato Research Institute of Weichang Manchu and Mongolian Autonomous County, Chengde, 068450 China

**Keywords:** *Alternaria solani*, Early blight, RNA-sequencing, Transcriptomic analysis

## Abstract

**Background:**

*Alternaria solani* (*A. solani*), the main pathogen of potato early blight, causes serious yield reductions every year. The application of fungicides is the most common and effective method of controlling *Alternaria*-caused diseases. The differentially expressed transcripts of *A. solani* infecting potato were identified, revealing a group of valuable candidate genes for a systematic analysis to increase the understanding of the molecular pathogenesis of *A. solani*, and providing scientific data for formulating additional measures to prevent and control potato early blight. In this study, a deep RNA-sequencing approach was applied to gain insights into *A. solani* pathogenesis. At 3, 4, and 5 days post inoculation (dpi), RNA samples from the susceptible potato cultivar Favorita infected with *A. solani* strain HWC-168, were sequenced and utilized for transcriptome analysis, and compared to the transcriptome obtained 0 dpi.

**Results:**

A total of 4430 (2167 upregulated, 2263 downregulated), 4736 (2312 upregulated, 2424 downregulated), and 5043 (2411 upregulated, 2632 downregulated) genes were differentially expressed 3, 4 and 5 dpi, respectively, compared with genes analysed at 0 dpi. KEGG enrichment analysis showed that genes involved in the pathways of amino acid metabolism, glucose metabolism, and enzyme activity were significantly differentially expressed at the late infection stage. Correspondingly, symptoms developed rapidly during the late stage of *A. solani* infection. In addition, a short time-series expression miner (STEM) assay was performed to analyse the gene expression patterns of *A. solani* and Profile 17 and 19 showed significant change trends 3, 4 and 5 dpi. Both profiles, but especially Profile 17, included enzymes, including transferases, oxidoreductases, hydrolases and carbohydrate-active enzymes (CAZYmes), which may play important roles in late fungal infection. Furthermore, possible candidate effectors were identified through the adopted pipelines, with 137 differentially expressed small secreted proteins identified, including some enzymes and proteins with unknown functions.

**Conclusions:**

Collectively, the data presented in this study show that amino acid metabolism, and glucose metabolism pathways, and specific pathway-related enzymes may be key putative pathogenic factors, and play important roles in late stage *A. solani* infection. These results contribute to a broader base of knowledge of *A. solani* pathogenesis in potato, as indicated by the transcriptional level analysis, and provide clues for determining the effectors of *A. solani* infection.

**Supplementary Information:**

The online version contains supplementary material available at 10.1186/s12870-023-04103-3.

## Introduction

Early blight of potato caused by *Alternaria solani* is one of the most important fungal diseases resulting in potato yield loss [1–3]. With the increasing demand for potatoes, the potato planting area has been increased continuously, and potatoes have become the fourth largest crop after wheat, rice, and corn. In recent years, the frequency early blight of potato incidences has increased, resulting in serious reductions in potato yield in China [4–6]. To date, the application of fungicides has been the most common and effective method of controlling *Alternaria* diseases in potatoes [7]. The long-term use of fungicides has resulted in the emergence of resistant *A. solani* strains in the field [8, 9]. Once these resistant strains become predominant, it will be impossible to control the disease with a single chemical fungicide. Therefore, it is necessary to systematically analyse and deeply understand the molecular pathogenesis of *A. solani*, to provide a scientific basis for the prevention and control of this disease.

Using genome-wide transcriptome analysis technology to analyse a host–pathogen interaction mechanism has led to significant progress in infectious diseases studies, and many possible candidate pathogenic factors have been thus identified. Thatcher analysed the transcriptome of alfalfa infected with *Fusarium oxysporum* and preliminarily identified 10 candidate effectors that may play important roles in *F. oxysporum* infection [10]. Using transcriptome sequencing, Barbara screened 58 candidate effectors that were highly expressed in rice blast [11]. Several upregulated proteins that are involved in amino acid synthesis, including methionine synthase and histidine synthase, were identified during the appressorium formation stage of phytophthora-infected potato [12]. Genes encoding enzymes for secondary metabolite synthesis including nonribosomal peptide synthase (NPS), polyketide synthase (PKS), terpene synthase (TPS), and cell wall-degrading enzymes (CWDEs), are required for pathogenicity or virulence and have been identified through comparative genomic analysis of *Alternaria brassicicola* [13].

However, transcriptome analyses based on the interaction between potato and *A. solani* have rarely been reported [14]. Only transcriptome analysis of potato interactions with *A. solani*, which was based observations taken from 1 to 48 h post inoculation has been reported, and the cell wall-degrading enzymes and metabolic processes may be important for *A. solani* infection of potato [14]. Furthermore, few reports have focused on the pathogenic effectors of *A. solani* in potato. In our previous study, the important effector proteins AsCEP112, AsCEP19 and AsCEP20 were identified [15, 16]. AsCEP112 is an important effector protein that targets host cell membranes and regulates host senescence-related genes to control host leaf cell senescence and chlorosis, and contributes to pathogen virulence [15]. The deletion of *AsCEP19* and *AsCEP20* from *A. solani* HWC-168 led to reduced virulence in potato leaves [16].

In our previous study, the entire genome of *A. solani* strain HWC-168 was sequenced (https://www.ncbi.nlm.nih.gov/nuccore/JRWV00000000.1/), which provided comprehensive gene annotations that have facilitated the accurate prediction of virulence-related genes in *A. solani* [17]. Therefore, to analyse the molecular pathogenesis of early blight of potato systematically and to gain an in-depth understanding of this infection, the transcriptome of the potato during interactions with *A. solani* from 0 to 3, 4 or 5 dpi was analysed. Candidate genes showing significant differential expression, indicating that they may be involved in the pathogenesis of *A. solani*, were identified. The results provide clues for determining the effectors of *A. solani* and help for establishing a broader knowledge of the pathogenic mechanism underlying *A. solani* infection of potatoes.

## Materials and methods

### The plant material and fungal isolate

The *A. solani* strain HWC-168 was isolated from the infected leaves of potatoes in Weichang County, Hebei Province in China. The potato cultivar Favorita is highly susceptible to early blight and was provided by the Inner Mongolia Grade Seed Potato Co., Ltd (Xinlin Gole, China).

### Plant growth and infection conditions

*A. solani* was cultured on potato dextrose agar (PDA) for strain activation and tomato juice agar medium (T4) to induce sporulation. The activated HWC-168 strain of *A. solani* was transferred to PDA plates from the PDA slant of the preserved strain and cultured at 25 °C in the dark for 3 days. Mycelial plugs (5 mm) were cut from the edge of a 3-day-old colony of the HWC-168 strain and transferred to a T4 medium plate. After 7 days of incubation at 25 °C in the dark, the active mycelia growing on the T4 plate were removed with sterile slide, and the T4 plate was irradiated with UV light (ultraviolet lamp power of 8 W) for 10 min. Finally, the T4 plate was cultured in the dark for alternating periods of 25 °C/12 h and 20 °C/12 h for 3 days [15]. When the T4 plate was covered with black spores, 2 ml sterile distilled water was added to wash the T4 plate, and the spores were collected, centrifuged at 10,000 rpm, and then diluted with sterile water to obtain a 10^6^ spores/mL suspension.

Potato (Favorita) was grown at 20 °C, for 16 h in the light and 8 h in the dark in a light incubator with 100% illumination for 2 weeks. A total of 30 potato plants were divided into 5 groups, with 6 plants in each group. For inoculation, 3 or 4 small wounds were made in each leaflet of 7-day-old potato plants and 6 μL of the 10^6^ spores/mL suspension was applied to each wound in the potato plants with four replicates established in 4 groups, and 6 μL of sterile distilled water was applied to each wound in one group, which was established as the negative control group. After inoculation, the potato seedlings were sealed, moistened and kept under the same growth conditions and disease progression was monitored. Then, 3, 4, and 5 days after inoculation, the leaves were collected for transcriptome sequencing.

### Total RNA extraction and high-throughput sequencing

The leaflet discs with a diameter of 9 mm were excised from the potato leaves infected by *A. solani* for 3, 4, and 5 days. Pure cultured spores from the 10^6^ spores/mL suspension collected 0 dpi were used as controls. A total of 4 groups of potato plants with four biological replicates were established. The 10^6^ spores/mL suspension was transferred to one 5.0-mL microcentrifuge tube for each group and snap-frozen in liquid nitrogen. For 4 groups of potato plants, three 9-mm-diameter leaf discs were cut from the leaves of each potato plant in each group 3, 4, and 5 dpi. A total of 18 leaf discs from each group were transferred to one 5.0-mL microcentrifuge tube and snap-frozen in liquid nitrogen. Samples were collected 0, 3, 4, and 5 dpi, immediately snap-frozen, and ground in liquid nitrogen prior to RNA extraction. Total RNA was extracted from the tissue using TRIzol Reagent (Plant RNA Purification Reagent for plant tissue) according to a method reported previously [18]. DNA was removed using DNase I (Takara). RNA quality was determined with a 2100 Bioanalyzer (Agilent) and quantified using an ND-2000 (NanoDrop Technologies). Only high-quality RNA (OD260/280 = 1.8 ~ 2.2, OD260/230 ≥ 2.0, RIN ≥ 6.5, 28S:18S ≥ 1.0, > 1 μg) was used to construct a sequencing library.

Using 1 μg of total RNA, the RNA-seq transcriptome library was prepared with the TruSeqTM RNA sample preparation kit from Illumina (San Diego, CA). Sequence reads (2 × 150 bp read length) were generated with an Illumina HiSeqxten/NovaSeq 6000 platform.

### Transcriptome data processing and analysis of differentially expressed genes

The transcriptomes of *A. solani* were analysed 3, 4, and 5 dpi, and compared to those at obtained 0 dpi. The raw paired-end reads were trimmed and quality controlled by SeqPrep (https://github.com/jstjohn/SeqPrep) and Sickle (https://github.com/najoshi/sickle) set with default parameters. Then clean reads were separately aligned to the *A. solani* reference genome (GCA_002837235.1, https://www.ncbi.nlm.nih.gov/assembly/GCA_002837235.1) in orientation mode using HISAT2 (v2.1.0, http://ccb.jhu.edu/software/hisat2/) software [19–21]. Due to the lack of annotations in the reference genome, the coding genes were first predicted using MAKER2 (v2.31.9) and then subjected to a BLASTX search against six common functional databases (NR/Swissport/GO/KEGG/EGGNOG/Pfam) and a pathogen-host interaction database (PHI-base; http://www.phi-base.org) (E value <  = 1e^−5^). Where available, a hit with the lowest E-score among the characterized genes was screened and functionally annotated. Then, the mapped reads of each sample were assembled and counted by StringTie (v1.3.3b, https://ccb.jhu.edu/software/stringtie/) using a reference-based approach [22]. The calculated raw expression value of each gene was normalized according to the fragments per kilobase of transcript per million fragments mapped (FPKM) method.

To identify the differentially expressed genes (DEGs) in the three different comparisons, the DESeq2 package (v1.24.0) in R software was utilized. Essentially, DEGs with an |log2 fold change|> 1 and a Q value ≤ 0.05 were considered to be significantly differentially expressed genes [23]. To better explore the expression pattern of the DEGs via three comparisons, the total DEGs with similar expression patterns in four multiple samples were clustered via Short Time-series Expression Miner (STEM) software. Profile with P values ≤ 0.05 were considered to be significant. In addition, a functional-enrichment analysis including KEGG enrichment analysis was carried out with KEGG (www.kegg.jp/kegg/kegg1.html) and KOBAS (http://kobas.cbi.pku.edu.cn/home.do) databases [24].

### *A. solani* secretome prediction and analysis pipeline

A pipeline used to predict the effectors of *A. solani* was developed. Effector proteins are considered to play important roles in the interaction between pathogenic fungi and plant hosts. Most effectors are secreted proteins. The prediction of secreted protein is usually based on the structural protein characteristics that include a N-terminal signal peptide but no transmembrane domain. In this study, subcellular localization software was also used to confirm that a protein was secreted outside cells. First, to obtain the protein sequence with an N-terminal signal peptide, the whole genome sequence of the HWC-168 strain of *A. solani* was carried out using signal-4.1 analysis software (http://www.cbs.dtu.dk/services/SignalP-3.0/) [25]. The analysis software of tmhmmv-2.0c (http://www.cbs.dtu.dk/services/TMHMM-2.0/) was used to identify and exclude proteins with a predicted transmembrane domain and to identify proteins containing an N-terminal signal peptide a without transmembrane domain [26]. Subsequently, the whole genome sequence HWC-168 was analysed with phobius 101 software (http://phobius.sbc.su.se/), to identify proteins predicted to carry a signal peptide without a transmembrane [27]. Finally, the predicted proteins obtained in the latter two steps were analysed with the analysis software ProtComp v3 (http://www.softberry.com/berry.phtml?topic=protcompan&group=help&subgroup=proloc), and the candidate proteins were analysed on the basis of whether they reside at a subcellular location or are secreted from the outer cell membrane [27].

### Validation of DEGs by RT-qPCR

To confirm the reliability of the data obtained by RNA-Seq, 10 DEGs, including *Caseinolytic Peptidase B* (*ClpB*), *Xyloglucan-specific endo-beta-1,4-glucanase A* (*XEG1*), *Glutathione S-transferase* (*GST*), *Cyanide hydratase* (*Cya*), *Pectin lyase 1* (*Pnl1*), *Melanin synthesis gene* (*BUF1*)*, Cutinase domain* (*CUTAD*), *Peptide transporter 2* (*Ptr2*), *Psi factor producing oxygenase A* (*PpoA*), and *Macrophage Expressed Gene 1* (*MPG1*) were randomly selected for RT-qPCR validation. The primers used in this experiment are listed in Table S1. RNA was reverse-transcribed into cDNA using a PrimeScript TM RT Master Mix Kit (TaKaRa) and real-time PCR was performed using a SYBR Premix Ex Taq II Kit (TaKaRa). Each sample was established with three replicates and the *elongation factor 1α (ef1α*) gene of *A. solani* was used as the reference. The relative expression levels of the different genes were calculated using the 2^−ΔΔCT^ method [28]. All data represent the standard deviation of four biological replicates.

## Results

### Progression of potato early blight disease

**Fig. 1:**
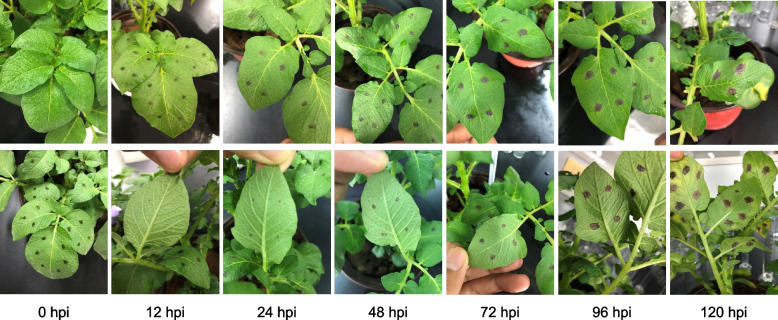
Pathological changes of potato leaf. Symptoms of Favorita cotyledons infected by HWC-168 0, 12, 24, 48, 72, 96, and 120 h post inoculation (hpi)

The leaves of potato Favorita were inoculated with *A. solani*. Twelve hours after inoculation, no obvious symptoms were found on either side of the leaf with the inoculation site. However, 24 h after inoculation, symptoms appeared on the back of the inoculated leaf. Within 72 h, a chlorotic ring around the inoculation site was evident, with obvious whorl symptoms and tissue collapse. Finally, the disease had spread rapidly by 96 and 120 h (Fig. 1).

### Preliminary analysis of the transcriptome sequencing data

**Fig. 2:**
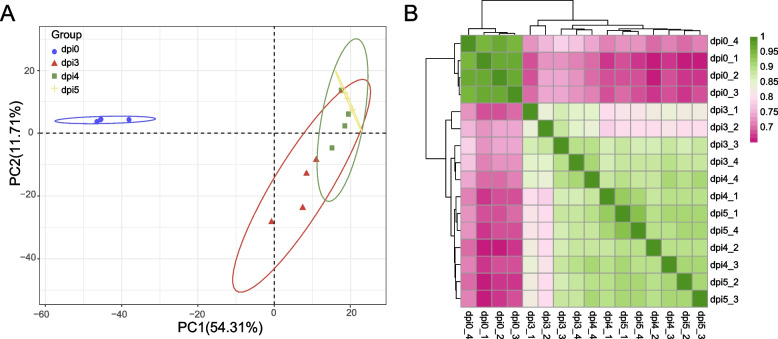
Global evaluation of RNA sequencing data obtained from in *A. solani* HWC-168. Principal component analysis (**A**) and heatmap showing differentially expressed genes (DEGs) (**B**). **A**. Principal component analysis (PCA) was performed to show the consistency between four biological replicates. The distance of each sample point represents the distance of the sample. The closer the distance is, the higher the similarity between the samples is. The horizontal axis represents the contribution of principal component 1 (PC1) to the identification of distinct samples, and the vertical axis represents the contribution of principal component 2 (PC2) to the identification of distinct samples; **B**. A correlation analysis was performed to determine the correlation between four biological replicates on the basis of Pearson correlation coefficients, and the numerical matrix is presented heatmap. The correlation is reflected by the colour change, and the colour depth represents the value of the Pearson correlation coefficients. The closer the Pearson correlation coefficients are to 1, the higher the similarity of the factors. The legend on the right shows the colour range of different Pearson correlation coefficients values. A sample cluster is shown on the left

RNA was prepared from leaf discs containing an inoculation site, and sampled 3, 4 and 5 dpi. RNA was extracted from pure cultured spores in the 10^6^ spores/mL suspension 0dpi as a control. To better understand the mechanisms of the interaction between *A. solani* and potato, we applied an Illumina HiSeqxten/NovaSeq 6000 RNA-seq approach to simultaneously assess the genome-wide expression profile of *A. solani* and potato at four different time points. After sequencing a total of 236.53 G clean data, approximately 13.41 G reads for each sample were used for subsequent analysis. For all libraries, the proportion of Q20 reached more than 96%, for Q30, it was not less than 92%, and GC content was 46.7% on average. The percentage of sequence reads mapped to the HWC-168 strain of the *A. solani* reference genome ranged from 95.36 to 95.56%, 1.19 to 17.14%, 2.84 to 5.61%, and 3.27 to 8.11% at 0, 3, 4, and 5 dpi, respectively (Table S2). The variability among samples was determined by preparing a heatmap of the sample-to-sample distance matrix based on Pearson correlation coefficients and principal component analysis (PCA) results [29, 30]. The data were analysed with the online tool of Majorbio Cloud Platform (https://cloud.majorbio.com/page/tools/). These results all revealed a clear distinction in the transcriptomes of *A. solani* at 3, 4, and 5 dpi, compared to that at 0 dpi. (Fig. 2A and Fig. 2 B).

### Identification of differentially expressed genes

**Fig. 3:**
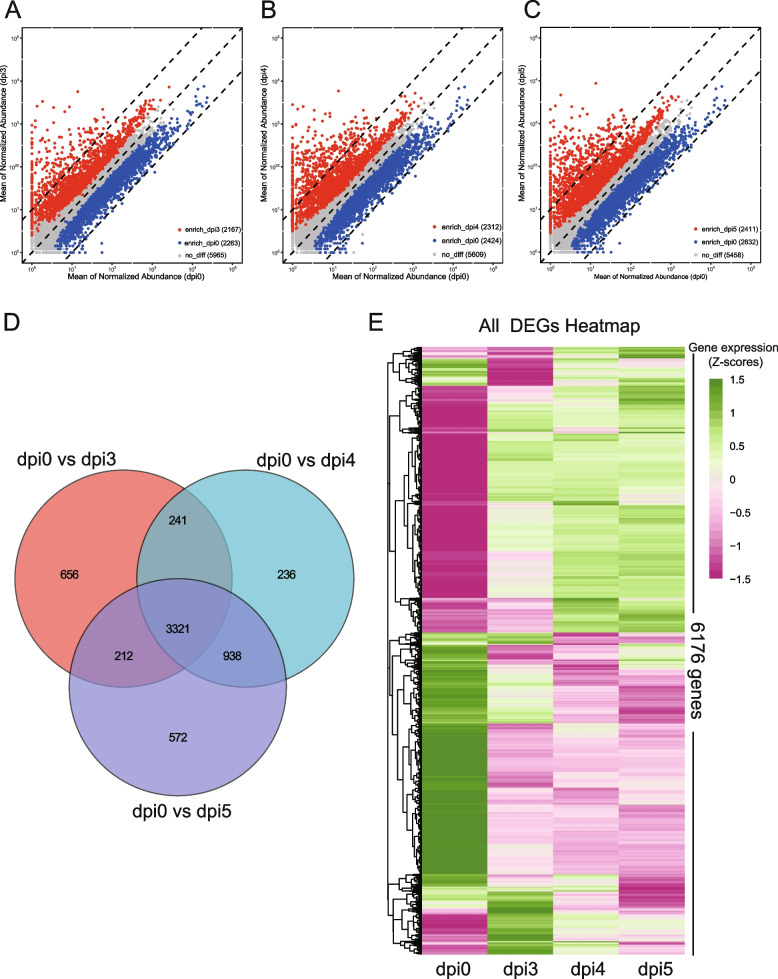
Summary of the identified differentially expressed genes (DEGs) during infection at different time points. **A**, **B**, and **C** Scatter charts showing differentially expressed genes 3, 4 and 5 days post inoculation (dpi) compared to that of the control 0 dpi, respectively. The abscissa represents the gene expression level at 0 dpi, and the ordinate represents the gene expression level 3, 4 and 5 dpi. A red dot indicates a significantly upregulated gene, a green dot indicates a significantly downregulated gene, and a gray dot indicates a gene for which the expression was not significantly different. D Venn diagram showing differentially expressed genes 3, 4 and 5 dpi compared to that of the control 0 dpi. The circles with different colours indicate different gene sets, and the values represent the number of common and unique genes among different gene sets; E Heatmap showing the DEGs. The colour in the figure indicates the expression level of the gene after standardization in each sample: red indicates that a gene with a high expression level, blue indicates a gene with a low expression level, and the number under the upper left colour bar indicates the specific expression level

In this study, the expression profiles of 11,217 predicted coding genes in *A solani* were used for further analysis, and the annotation information of these genes and relative expression levels in all samples are presented in Table S3. Genes with significant changes in expression were identified by pairwise comparison between infection times (3 vs. 0 dpi, 4 vs. 0 dpi, 5 vs. 0 dpi). The total numbers of DEGs in these comparisons were 4430 (2167 upregulated, 2263 downregulated), 4736 (2312 upregulated, 2424 downregulated), and 5043 (2411 upregulated, 2632 downregulated) (Fig. 3A, Fig. 3B and Fig. 3C) DEGs, respectively. Among these DEGs, 3321 genes were found to be common (Fig. 3D), as detailed in Table S4. Additionally, a total of 6176 DEGs were differentially expressed at one or more time points. The gene expression heatmap for all DEGs is shown (Fig. 3E).

### Short time-series expression miner

**Fig. 4:**
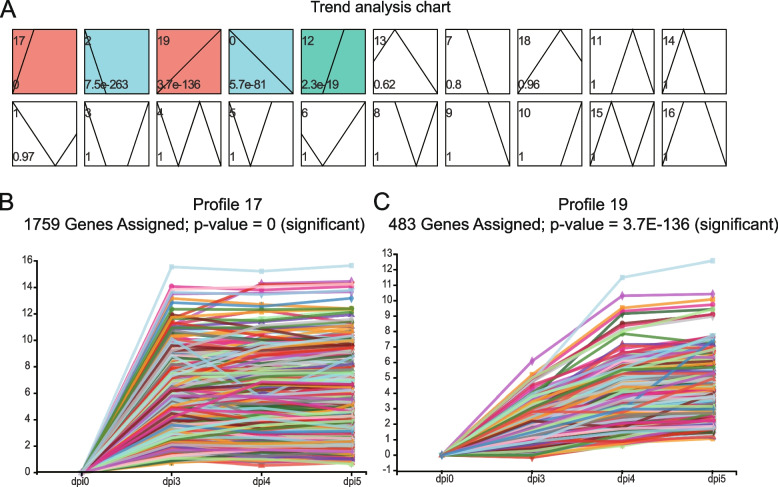
Short time-series expression miner (STEM) analysis of the gene expression pattern. **A**. STEM software was used to analyse the gene expression pattern, and twenty profiles exhibited significant clustering of gene expression patterns. The black line represents the general tendency in each profile, and profile with the same colour belong to the same profile; **B** and **C**. The changes of all genes included in Profile 17 and 19, respectively. The number of unigenes in each profile is labelled above the plot

The molecular mechanism of pathogenic fungi infection of host plants and the mechanisms causing disease symptoms involve complicated biological processes that are regulated by multiple genes, and those with the same expression pattern may function in the same biological process [31]. In this study, to gain insights into the dynamic changes in gene expression during development, short time-series expression miner (STEM) software was used to categorize the identified DEGs in the 3 vs. 0 dpi, 4 vs. 0 dpi, and 5 vs. 0 dpi comparisons into a pattern based on 20 profiles (Fig. 4A). The pattern analysis results showed that five profiles exhibited significant gene expression patterns, with Profile 17 and 19 upregulated, and profile 2 and 0 downregulated, compared relative to that at 0 dpi. Consistent with background research results, the symptoms at inoculation site were more obvious after 72 h after inoculation. Therefore, we mainly focused on and searched for DEGs in key metabolic pathways that were expressed at high levels in the late infection stage. Two interesting regulatory patterns, Profile 17 and 19, with 1759 genes and 483 genes in Profiles 17 and 19, respectively, were selected to further understand the corresponding functions in detail (Fig. 4B, Fig. 4C, and Table S5).

### KEGG metabolic pathway enrichment analysis

A Kyoto Encyclopedia of Genes and Genomes (KEGG) enrichment analysis showed that upregulated DEGs were mainly related to glucose metabolism, amino acid metabolism, and lipid metabolism, including TCA cycle, pyruvate metabolism, galactose metabolism, beta-alanine metabolism, pentose and glucuronate interconversions, valine, leucine and isoleucine biosynthesis and degradation, while the downregulated DEGs were mainly related to signal transduction and immune response, including prodigiosin biosynthesis, protein processing in the endoplasmic reticulum, ubiquitin-mediated proteolysis and basal transcription factors (Table 1) [32–34].

**Table 1:** KEGG enrichment of three stages following infection

Time		Description	Number	Pvalue
0v3	up	Ribosome	69	1.27 × 10^–18^
		Valine, leucine and isoleucine biosynthesis	11	6.84 × 10^05^
		Lysine biosynthesis	9	1.99 × 10^–4^
		Citrate cycle (TCA cycle)	16	3.14 × 10^–4^
		Pyruvate metabolism	21	8.88 × 10^–4^
	down	Prodigiosin biosynthesis	5	7.37 × 10^–5^
		Linoleic acid metabolism	3	3.33 × 10^–3^
		Protein processing in endoplasmic reticulum	23	2.113 × 10^–3^
		Longevity regulating pathway—multiple species	11	1.439 × 10^–3^
		Ubiquitin mediated proteolysis	18	3.151 × 10^–3^
0v4	up	Ribosome	56	2.05 × 10^–11^
		Valine, leucine and isoleucine degradation	26	1.55 × 10^–6^
		Galactose metabolism	20	3.22 × 10^–5^
		beta-Alanine metabolism	17	8.22 × 10^–5^
		Pentose and glucuronate interconversions	18	6.03 × 10^–5^
	down	Prodigiosin biosynthesis	5	2.048 × 10^–4^
		Mitophagy—yeast	13	3.6298 × 10^–3^
		Necroptosis	10	3.088 × 10^–3^
		Ubiquitin mediated proteolysis	21	2.3468 × 10^–3^
		Fatty acid biosynthesis	6	7.4068 × 10^–3^
0v5	up	Ribosome	64	2.078 × 10^–15^
		Galactose metabolism	22	4.088 × 10^–6^
		Starch and sucrose metabolism	33	8.518 × 10^–6^
		Valine, leucine and isoleucine degradation	25	2.028 × 10^–5^
		N-Glycan biosynthesis	20	8.18 × 10^–5^
	down	Prodigiosin biosynthesis	5	3.088 × 10^–4^
		Mitophagy—yeast	15	7.498 × 10^–4^
		Basal transcription factors	13	2.858 × 10^–3^
		Ubiquitin mediated proteolysis	21	6.528 × 10^–3^
		Linoleic acid metabolism	3	7.8518 × 10^–3^

**Fig. 5:**
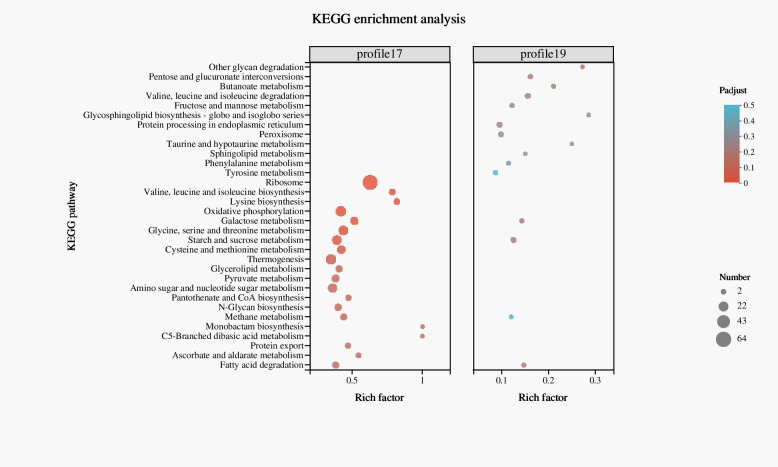
**A** Kyoto Encyclopedia of Genes and Genomes (KEGG) enrichment analysis of Profile 17 and 19. The abscissa axis represents the enrichment factor, which is the ratio of the number of genes enriched in a pathway to the annotation gene number, and the ordinate axis indicates the pathway name. The size of the dot indicates the number of genes in the pathway, and the colour of the dot corresponds to different p-adjusted ranges

Ribosome, valine, leucine and isoleucine biosynthesis, lysine biosynthesis, oxidative phosphorylation, and galactose metabolism in Profile 17 and other glycan degradation, Galactose metabolism in Profile 19 were significantly enriched, suggesting that these metabolic pathways may be involved in the pathogenesis of *A. solani* (Fig. 5). In these profiles, 579 enzymes were identified, including 128 oxidoreductases, 140 transferases, 176 hydrolases and 205 carbohydrate-active enzymes (CAZYmes), as well as necrosis-and ethylene-inducing protein-like protein 1 precursor, and glycine hydroxymethyl transferase, which showed a significant increase in expression 3 dpi. In addition, genes encoding enzymes, including the DUF1929-domain-containing protein, copper/zinc binding superoxide dismutase, cutinase-domain-containing protein, and hydrophobin-like protein, the expression of was consistently increased 3, 4, and 5 dpi, were also identified in these profiles (Table 2).

**Table 2:** Key genes identified in STEM

Profile	Gene Number	Gene Name	Description	Log2 Fold Change		
				0v3	0v4	0v5
17	gene08293	*LysC*	bifunctional aspartokinase/homoserine dehydrogenase	3.11*	2.23*	2.00*
17	gene04746	*GalA*	glycoside hydrolase family 36 protein	4.17*	4.73*	5.03*
17	gene00763	*GlyA*	glycine hydroxymethyltransferase	1.94*	1.03	0.91
17	gene05992	*MetX*	homoserine O-acetyltransferase	2.08*	1.79	1.63*
17	gene05496	*GLO1*	DUF1929-domain-containing protein	1.96*	2.01*	2.33*
17	gene04945	*AroC*	chorismate mutase	3.96*	3.48*	3.47*
17	gene10711	*MPG1*	hydrophobin-like protein	7.10*	8.85*	9.41*
17	gene04030	*SOD1*	copper/zinc binding superoxide dismutase	3.37*	4.28*	4.61*
17	gene03396	*NLP*	necrosis-and ethylene-inducing protein-like protein 1 precursor	1.97*	1.21	1.71*
17	gene01526	*XEG1*	endoglucanase A precursor	4.42*	6.05*	6.31*
17	gene03386	*PGX1*	polygalacturonase precursor	3.61*	4.65*	4.88*
17	gene05303	*BUF1*	NAD(P)-binding protein	2.17*	3.11*	3.04*
17	gene09458	*BglX*	beta-glucosidase-like protein	5.89*	6.94*	6.99*
17	gene06670	*CUTAD*	cutinase-domain-containing protein	11.60*	14.18*	14.23*
19	gene05108	*Pnl1*	pectin lyase-like protein	3.90*	4.48*	6.07*
19	gene03176	*Pel*	pectate lyase-like protein	1.58	4.70*	6.42*

^*^represent significant change at the time point

### *A. solani* effector prediction

**Fig. 6:**
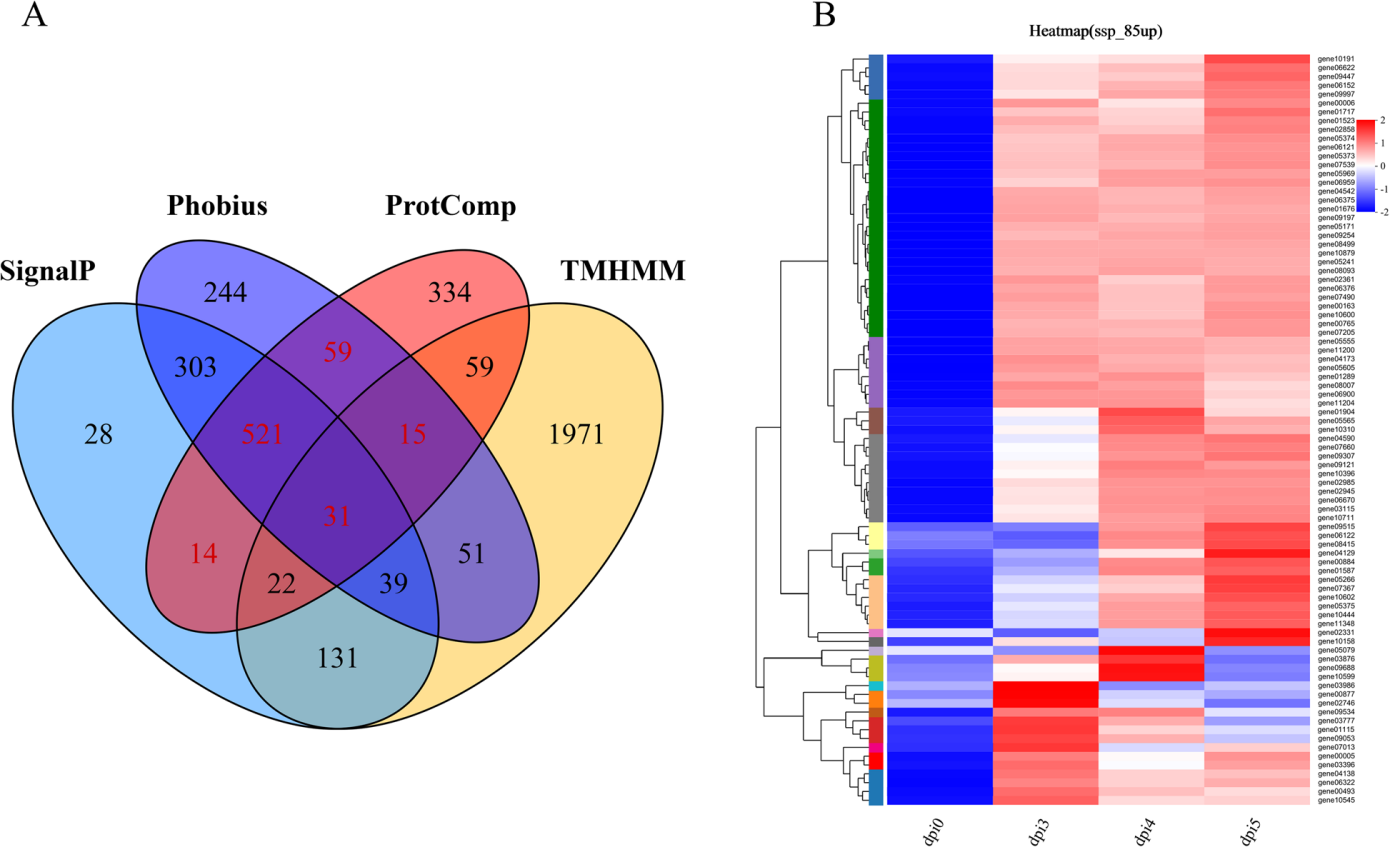
Pipelines used to predict effectors of *A. solani* infection*.* **A**. Venn diagram showing the predicted number of secreted proteins. The circles with different colours indicate the results predicted by different analysis software; signalp indicates a protein containing an N-terminal signal peptide as predicted by signalp-4.1; tmhmm indicates a tmhmm-2.0c prediction of proteins containing transmembrane domain(s); phobius indicates phobius 101 predicted protein carrying an N-terminal signal peptide but no transmembrane domain; and protComp indicates Protcomp v3 prediction of proteins with a subcellular location or that was secreted from the outer cell membrane. The number of proteins predicted by ProtComp that that were also predicted signalp or phobius but not by tmhmm, was predicted to be a secretory protein. The red numbers indicated predicted secreted proteins. **B**. Heatmap of 85 SSP genes after an expression pattern cluster analysis. The colour in the figure indicates the expression level of a gene after standardization in each sample: red indicates gene expressed at a high level, blue indicates a gene expressed at a low level, and the number under the upper left colour bar indicates the specific expression level

According to the genome information of *A. solani* strain HWC-168 [17], we adopted the pipeline to search for *A. solani* candidate effectors and identified those with features common to effectors (Fig. 6) [35, 36]. In total, 640 secreted proteins (SPs) were identified using the analysis software programs of signal-4.1, tmhmmv-2.0c, phobius 101 and ProtComp v3, which were adopted to analyse the whole-genome sequence of the HWC-168 strain of *A. solani* (Fig. 6A). The SPs identified by the pipelines included 275 small secreted proteins (SSPs) (< 300 amino acids). Among the 275 genes encoding SSPs, 137 genes showed significant differential expression 3, 4, or 5 dpi. A total of 137 SSPs may play important roles in the infection of potato by *A. solani*. In addition, 85 of 137 SSPs genes were upregulated 3, 4, or 5 dpi (Fig. 6B).

The candidate effectors of a large number of enzymes, including pectate lyase, cutinases, xylanase, hydrophobin-like protein, and glycoside hydrolase were obtained, and 85 SSPs were compared via the PHI-base database (http://www.phi-base.org/) and the CAZY database (http://www.cazy.org/) (Table 3). These candidate effectors may play important roles in the later stage of *A. solani* infection of potatoes.

**Table 3:** Candidate effectors of *A. solani* during infecting potatoes

Gene name	Gene number	Protein size	Cysteine number	Description	PHI ID	Pathogen Species	CAZy Family
*PL1332*	gene02361	243	10	pectate lyase	PHI:4845	*Alternaria brassicicola*	*Polysaccharide Lyases3-2*
*PELDP*	gene02858	273	10	pectate lyase precursor	PHI:180	*Nectria haematococca*	Polysaccharide Lyases3-2
*NLP2*	gene03396	235	2	necrosis-and ethylene-inducing protein-like protein 1 precursor	PHI:2712	*Verticillium dahliae*	-^d^
*4LysM*	gene06322	197	6	hypothetical protein	PHI:6831	*Verticillium dahliae*	-
*CUTAD*	gene06670	250	4	cutinase-domain-containing protein	PHI:69	*Botrytis cinerea*	Carbohydrate Esterases-5
*OB015*	gene10191	247	4	glycoside hydrolase	PHI:1575	*Fusarium graminearum*	Auxiliary Activities 9
*PELDL*	gene10444	236	14	pectate lyase-like protein	PHI:180	*Nectria haematococca*	Polysaccharide Lyases3-2
*PELD*	gene10879	282	10	pectate lyase	PHI:180	*Nectria haematococca*	Polysaccharide Lyases3-2
*CfmB*	gene11200	208	8	CFEM-domain-containing protein	PHI:4016	*Aspergillus fumigatus*	-
*Caf1*	gene02985	207	1	secretory pathway protein-like protein Ssp120	PHI:3936	*Sclerotinia sclerotiorum*	-
*XYNA*	gene03115	235	2	endo-1,4-beta-xylanase I precursor	-^b^	-^c^	Glycoside Hydrolases11
-^a^	gene04129	171	4	hypothetical protein	-	-	Carbohydrate-Binding Modules 13
*CUTA*	gene05266	226	5	cutinase	-	-	Carbohydrate Esterases5
-	gene07013	271	2	concanavalin A-like lectin/glucanase	-	-	Glycoside Hydrolases12
-	gene07205	269	3	hypothetical protein	-	-	Glycoside Hydrolases128
-	gene07367	229	6	glycoside hydrolase	-	-	Auxiliary Activities 9
-	gene07490	144	6	hypothetical protein	-	-	Carbohydrate-Binding Modules 50
*EXLX*	gene08415	226	6	barwin-like endoglucanase	-	-	Carbohydrate-Binding Modules 63
-	gene09447	221	3	hypothetical protein	-	-	Auxiliary Activities 9
-	gene10396	272	15	endoglucanase	-	-	Glycoside Hydrolases 45

^a^ Dashes indicate that the gene has no name

^b^ and ^c^ Dashes indicate that the protein has no found in the PHI-base database

^d^ Dashes indicate that the protein has no found in the CAZY database

### RT-qPCR Validation

**Fig. 7:**
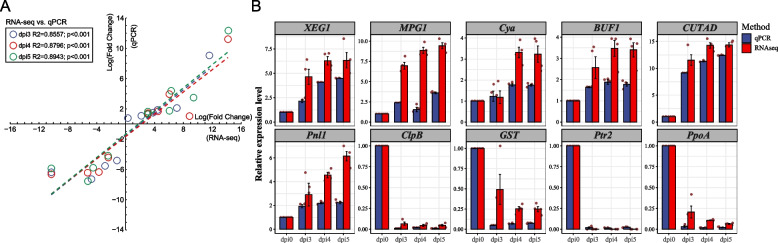
Validation of the expression of candidate genes during infection as determined by quantitative real-time RT-PCR analysis. A. Correlation analysis of 10 randomly selected differentially expressed genes (DEGs) based on RNA-seq and qRT-PCR data. The relative expression values are reported after log2 transformation. B. Gene *ClpB*, *XEG1*, *GST*, *Cya*, *Pnl1*, *BUF1*, *CUTAD*, *Ptr2*, *PpoA* and *MPG1* were subjected to RT-qPCR. The relative expression values are reported after log2(x + 1) transformation

To validate the reliability of the RNA-Seq data, ten randomly selected DEGs involved in the infection pathway were selected for RT-qPCR analysis. The qPCR expression patterns of the DEGs were found to be consistent with the RNA-Seq analysis results at in different developmental stages (dpi3, R^2^ = 0.8887; dpi4, R^2^ = 0.8796; dpi5, R^2^ = 0.8943), which confirmed the reliability and accuracy of the RNA-Seq data (Fig. 7A). Moreover, a bar plot comparing the differences in the expression of the corresponding gene expression identified via RNA-Seq RT-qPCR is shown in detail on the right side of Fig. 7B.

## Discussion

In recent years, studies associated with *Alternaria* spp. have focused primarily on plant responses to infection, the genes encoding proteins that interact between *A. solani* and its potato host have received little attention [13]. *A. solani* infection progression through a 48 time courseh has been reported [14]. At 24 h post inoculation of *A. solani* infection into a potato leaf, necrosis of the first epidermal cells on the adaxial side of the leaf has been observed. Similar to our study, symptoms appeared on the backside of a leaf that had been inoculated 24 h. However, Brouwer studied *A. solani* infection of potato leaves only for the first 48 h, and the interaction of genes in the late stage of infection was not clear [14]. A transcriptome analysis of the genes in potato and *A. solani* that exhibited interactions in the early infection stage showed that cell wall-degrading enzymes and metabolic processes may be important for early *A. solani* infection of potato [14]. In our previous study, we reported that the effector proteins AsCEP112, AsCEP19 and AsCEP20, and play important roles in late-stage *A. solani* infection of potato leaves [15, 16]. In the current study, we inoculated potato leaves with *A. solani* and found that near the inoculation site, there were obvious whorl symptoms and tissue collapse within 72 h, and the disease spread rapidly between 96 and 120 h. Therefore, we focused on protein interaction in the late stage of infection, which was further investigated by genome-wide transcriptomic analyses. By performing studies at the molecular level, we aimed to provide a theoretical basis for the preliminary elucidation of the pathogenic mechanism underlying *A. solani* infection.

The transcriptome data showed that a large number of the DEGs were expressed, at as determined by comparing expression levels at 3 vs. 0 dpi, 4 vs. 0 dpi, and 5 vs. 0 dpi, and 54% (3321/6176) of these DEGs were common to the comparison groups. The number of DEGs of the late infection stage was significantly higher than that the early infection stage [14]. Through a KEGG enrichment analysis, we found that the activation of pathways involved in sugar, starch, and protein metabolism, including many proteins and enzymes, was significantly upregulated in the three infection stages.

In addition, the DEGs were analysed by STEM software, which showed that the expression of a large number of enzymes in Profile 17 and 19 were significantly upregulated, including kinases and dehydrogenases, glycoside hydrolase family proteins, peroxidases, and transferases. These enzymes may be related to pathogenicity. Chorismate mutase (AroC) has been reported to inhibit SA-induced immune responses [37]. Peroxide dismutase (SOD1) is involved in inhibiting the oxidative damage of pathogens and plant resistance [31]. Glycoside hydrolases are involved in degrading the cell wall of plants [38–42]. Furthermore, Mpg1 and BUF are related to appressorium formation and invasion in *Magnaporthe grisea* [43, 44].

Through the pipelines, we found that 137 SSPs shared features common to effectors that were highly differentially expressed during the late stage of infection, making these SSPs possible pathogenic factor candidates. A large number of enzymes among these SSPs, including pectate lyase, cutinase, xylanase, hydrophobin-like protein, glycoside hydrolase, necrosis and ethylene-inducing peptide 1 (Nep1)-like proteins (NLPs) were identified [45, 46]. These SSPs exhibit high homology with the proteins encoded by genes that have been shown to be associated with pathogenicity. This finding indicates that the enzymes involved in the late stage of infection may be the key factors in pathogenesis. We also found many proteins with unknown functions that are expected to provide clues for the subsequent determination of pathogenic factors.

## Conclusions

In this study, deep RNA-sequencing was applied to understand the pathogenic mechanism of *A. solani* infection of potato in vivo. Based on a transcriptome analysis, a total of 3683, 4097, and 4396 DEGs were identified in 3 vs. 0 dpi, 4 vs. 0 dpi, and 5 vs. 0 dpi comparisons, respectively. An enrichment analysis showed that the genes involved in amino acid metabolism, glucose metabolism, and enzyme activity were significantly expressed at the late stage of infection. By performing short time-series expression miner (STEM) analysis, we found a large number of enzyme proteins, including transferases, oxidoreductases, hydrolases, and CAZYmes, which may play important roles in the late stage of fungal infection. We also identified possible candidate effectors using adapted pipelines and found 137 differentially expressed small secreted proteins including several enzymes. These results provide clues for determining the effectors in *A. solani* and contribute to the broad knowledge of the pathogenic mechanism underlying *A. solani* infection of potato at the transcriptional level.

## Supplementary Information

**Additional file 1:** **Table S1.** Primers used for real time PCR.

**Additional file 2: Table S2.** Summary of transcriptome sequencing datasets.

**Additional file 3: Table S3.** The annotated results of all predicated coding genes and corrsponding expression level in all samples.

**Additional file 4: Table S4.** Commonly expressed and uniquely expressed genes at different points.

**Additional file 5: Table S5.** The expression level change of genes associated to Profile 17 and 19.

## Data Availability

All datasets generated for this study are included in the manuscript/Supplementary Files. (Accession: CRA008628) (https://ngdc.cncb.ac.cn/gsa/).

## Abbreviations

Aroc: Chorismate mutase
Bcsod1: Peroxide dismutase
CAZYmes: Carbohydrate-active enzymes
CWDEs: Cell wall degrading enzymes
dpi: Days post inoculation
DEGs: Differentially expressed genes
FPKM: Per Million fragments mapped
KEGG: Kyoto encyclopedia of genes and genomes
NLPs: Nep1-like proteins
NPS: Non-ribosomal peptide synthase
PCA: Principal component analysis
PDA: Potato dextrose agar
PKS: Polyketide synthase
SSPs: Small secreted proteins
STEM: Short time-series expression miner
TPS: Terpene synthase
